# Observations on Vaccination and Small Pox, &c.

**Published:** 1841-08

**Authors:** George Gregory

**Affiliations:** Physician to the Small Pox Hospital


*Observations on Vaccination and Small Pox, more especially with reference to the theory of Vaccine Influence, and the relation subsisting between the Cicatrix and the character of the Consecutive Variola.* By George Gregory, M. D., Physician to the Small Pox Hospital.—The observations of the author in the present paper are intended to point out, first, the alarming increase of small pox in the metropolis, as shown by the books of the Small Pox Hospital; and, secondly, the insufficiency of the appearance of the cicatrix of the former vaccination as a test of the degree of protection afforded by the process.

Upon the first point he adduces the fact, that the admissions in the first three quarters of 1840 only amounted to one hundred and forty two, being at the rate of sixteen per mensem, while in the twenty-five days immediately preceding the reading of the paper, they amounted to ninety-three being at the rate of nearly four per diem, a greater number than was ever admitted in one month since the establishment of the hospital in 1746. Of three hundred and sixteen cases admitted in 1840, one hundred and ninety-four had not been vaccinated, of whom eighty-seven died, or forty-five per cent! one hundred and twenty had been vaccinated, of whom only eight died, being at the rate of seven per cent; the remaining two had had the small pox previouly. Of the three hundred and sixteen patients, forty-seven were under five years of age, of whom twenty-eight died; forty-five between five and fifteen, of whom nine died; two hundred and twenty-four were adults, of whom fifty-eight died. The total mortality was ninety-five, or thirty per cent. on the gross admissions. With reference to the second point, the author entered at some length into an explanation of the causes by which the many observed varieties in the appearance of the cicatrix may be explained; and presented the society with two series of well-marked cases, in the first of which severe small pox occurred in cases presenting perfectly normal cicatrices; whilst in the second, the opposite anomaly presented itself, the lightest and truly varicelloid eruptions co-existing with small and very imperfect cicatrices. In the conclusion of this paper, the author expresses a doubt of the conclusion seemingly derived from the late experiments of Mr. Ceeley, of Aylesbury, of the identity of the vaccine and variolous poisons.

Various questions were put to Dr. Gregory after the reading of his very interesting paper, having reference to vaccination and variola. The more interesting of the points commented upon were, first, the question of the severity of the present epidemic; secondly, the proper period of performing vaccination; thirdly, the ages at which patients affected with small pox after vaccination were vaccinated; fourthly, re-vaccination; and, fifthly, the new lymph, or variola-vaccina.

As to the first, Dr. Webster contended that small pox was not at the present time so general as Dr. Gregory's paper would lead us to believe. He compared the mortality of 1838, the year of the last epidemic, with that of the year 1840, and showed by the tables of the registrar-general, that the mortality in the former was two-thirds more than the mortality of the latter year.

Dr. Gregory explained that the present epidemic did not commence until October, 1840; that of 1838 commenced in the same month, 1837; Dr. Webster would not arrive at a proper conclusion unless he limited his calculations to the last three months of 1840, and compared the deaths occurring in them with those taking place in the same months of 1837. It was known that epidemics required six months to reach their height, and as long a period to recede; that of 1837 reached its height in May, 1838; he expected the present would be most prevalent in May or June of the present year. Under all circumstances, he believed that the present epidemic was not so general as that of 1838. The Small Pox Hospital's entries and deaths had been found during the last thirty years always to bear a direct relation to the number of cases of, and death from small pox throughout London, and he considered that they afforded a sufficient field on which to found a statistical inquiry. On the second point, Dr. Gregory observed, that so long as the vaccine vesicle had all the usual characteristics, and there was sufficient constitutional disturbance, it mattered little as to when vaccination was performed, whether in the first month, or the first year of life. The difficulty of vaccinating very young children did not depend upon want of susceptibility in the patient, but on the absence of that plumpness which was necessary to be present for the proper performance of vaccination. About the fourth month, this plumpness being usually present, was an excellent time to perform the operation.

Mr. Ceely agreed with the statements of Dr. Gregory, but believed that vaccination might be properly performed in children at the very earliest age, if the skin were merely scratched with a lancet, instead of an attempt being made to insert the lymph by means of puncturing the skin. To answer the third question there was much difficulty; all the information that could be gained from patients being merely, that they were vaccinated in early life. Dr. Gregory had noticed, however, that more cases of small pox had followed vaccination when performed at the adult period, than when it had been effected in infancy. He thought this showed that infancy was the proper period for vaccination to be performed in, and that the effects of the operation were more decided at this period of life than at the adult period, because in the former a less mass of fluid was required to be impregnated with the protective influence of the virus. The fourth month being a time when the constitution was not affected by dentition, or other contending influences, was the best period to vaccinate. As to re-vaccination, he had little experience in the matter; but this he had observed in a family re-vaccinated under his own eye,—that neither the age of the patient, nor the appearance of the cicatrix upon the arm, appeared to have had any effect upon the vaccination; in some there was a mere papular eruption; in others, the vesicle went through its usual course. On the fifth point, Dr. Gregory made inquiry of members whether or no they had used the new lymyh—the variola-vaccine, and if so, with what results? Was it in any way different from the lymph which had been in constant use from the time of Jenner to the present? For his own part he believed it was less certain in its results, and in this respect bore a great analogy to the small pox itself. He had found, for instance, that there was no certainty in the kind of vesicle it would produce; in one person it would be mild, in another irritable; as in small pox we found the confluent sometimes produced mere varioloid disease, and mere varioloid disease sometimes the most malignant kinds of small pox. Mr. Ceeley believed that there was no material difference in the two kinds of lymph: the varieties observed in its effects being dependent upon the soil into whch it was inserted, and the season in which it was used.—*Trans. Roy. Med and Chirurg. Soc., in Prov. Med. and Surg. Journ.*

